# The study of hidden habitats sheds light on poorly known taxa: spiders of the Mesovoid Shallow Substratum

**DOI:** 10.3897/zookeys.841.33271

**Published:** 2019-04-23

**Authors:** Enrique Ledesma, Alberto Jiménez-Valverde, Alberto de Castro, Pablo Aguado-Aranda, Vicente M. Ortuño

**Affiliations:** 1 Research Team on Soil Biology and Subterranean Ecosystems, Department of Life Science, Faculty of Science, University of Alcalá, Alcalá de Henares, Madrid, Spain University of Alcalá Madrid Spain; 2 Entomology Department, Aranzadi Science Society, Donostia - San Sebastián, Gipuzkoa, Spain Aranzadi Science Society San Sebastián Spain

**Keywords:** Araneae, Iberian Peninsula, inventory completeness, species distributions, stone debris

## Abstract

The scarce and biased knowledge about the diversity and distribution of Araneae species in the Iberian Peninsula is accentuated in poorly known habitats such as the Mesovoid Shallow Substratum (MSS). The aim of this study was to characterize the spiders inventory of the colluvial MSS of the Sierra de Guadarrama National Park, and to assess the importance of this habitat for the conservation of the taxon.

Thirty-three localities were selected across the high peaks of the Guadarrama mountain range and they were sampled for a year using subterranean traps specially designed to capture arthropods in the MSS. Species accumulation curves were built both for the observed species richness and for the non-parametric richness estimators. The literature was reviewed in order to update the distributional maps of the rarest species.

Forty-two species were collected, of which four were species new to science. More than half were represented by one or two individuals which caused the accumulation curves to rise slowly and to end without reaching an asymptote. Almost half of the species showed significant increases in their Iberian distribution ranges. Two species were recorded for the first time in the Iberian Peninsula and 32 species were new additions to the spider checklist of the Sierra de Guadarrama National Park.

## Introduction

The Mesovoid Shallow Substratum (MSS) is a subterranean habitat originally described by Juberthie et al. (1980, 1981) and Uéno (1980, 1981) as the network of voids and interstices found just above the deep subterranean domain and immediately beneath the soil (if soil exists). The structure of the MSS is a direct consequence of the action of different lithological processes in diverse substrates; accordingly, various types of MSS have been categorised, including colluvial, alluvial, volcanic, and bedrock (see Mammola et al. 2016). The absence of light, the usually high and constant relative humidity and the cushioned temperature fluctuations throughout the year are common abiotic conditions for any MSS (Mammola et al. 2016; Giachino and Vailati 2010; Pipan et al. 2011; Mammola et al. 2017a). The shallow condition of the MSS typically results in a significant flow of material and energy from the upper layers (Gers 1998).

The phylum Arthropoda dominates in the MSS (Mammola et al. 2017a; Nitzu et al. 2010, 2014; Ortuño et al. 2013; Langourov et al. 2014; Jiménez-Valverde et al. 2015). In general, the MSS assemblages are very rich and diverse and are formed both by hypogean species with different degrees of adaptation to the subterranean environment and by a notable contingent of epigean and endogean species which transit between the surface and the hypogean environment (Gers 1998; Pipan et al. 2011; Pipan and Culver 2012; Nitzu et al. 2014; Jiménez-Valverde et al. 2015; Rendoš et al. 2016; Mammola et al. 2017a). The MSS, by virtue of its biophysical characteristics, plays a fundamental ecological role as an ecotone between the surface and the deep subterranean environment (Moseley 2010), as well as serving as a biogeographic corridor and climatic refuge (Ortuño et al. 2013; Moseley 2010; Růžička 1993; Hernando et al. 1999; Růžička et al. 2012). All of these roles have obvious important implications in conservation.

The study of the MSS is limited by logistical difficulties and requires substantial research effort. Consequently, knowledge about MSS biodiversity is scarce and tends to be geographically biased (Jiménez-Valverde et al. 2015; Mammola et al. 2016). However, when the sampling difficulties can be overcome, exploration of the MSS usually reveals taxonomic novelties and interesting species records (see Mammola et al. 2016, for an extensive list of references). In the Iberian Peninsula, for example, new species from different arthropod orders have been described from the MSS, including the Orthoptera (Barranco et al. 2013), Coleoptera (Toribio and Rodríguez 1997; Carabajal et al. 1999; Faille et al. 2012; Ortuño et al. 2014), Collembola (Baquero et al. 2017), Diplura (Sendra et al. 2017), and Diplopoda (Gilgado et al. 2015a, 2015b, 2017; Akkari et al. 2018). Therefore, the MSS is a habitat that hosts many rare and poorly known species (see, for instance, Ortuño 1996, 2002, 2004; Ortuño and Toribio 1994; Ortuño and Martínez-Pérez 2011; Ortuño et al. 2014; Gilgado et al. 2015a, 2015b, 2015c; Jiménez-Valverde et al. 2015). However, the significance of the potential of the MSS to enhance our knowledge of biodiversity is not easy to evaluate because of the minimal number of comprehensive studies that have covered a broad spatial, temporal, and taxonomic scale.

Araneae is a hyperdiverse taxon that includes more than 47200 accepted species distributed in 116 families and in more than 4000 genera (World Spider Catalog 2018). Approximately 1000 spider species across 48 families occur in subterranean ecosystems (Mammola and Isaia 2017). Nevertheless, the taxonomic and chorologic knowledge on spiders is generally scarce and geographically biased, despite their high abundance and richness in almost all ecosystems, their key role in ecological networks, and their potential as biological indicators (New 1999). The Iberian-Balearic region harbours around 1382 species (Morano et al. 2014), although the true number is estimated to lie between 1500 and 2000 species (Melic et al. 2015). Given the current state of knowledge, around 19% of the species are considered Iberian endemics (Melic 2001).

The paucity of knowledge about Iberian spiders is illustrated by the fact that 20% of the species are known from just a single record and 50% of the species from fewer than 5 records (Cardoso and Morano 2010). The accumulated records also show a strong geographical bias, as illustrated by the fact that some provinces have fewer than 30 recorded species (Morano et al. 2014). Approximately one hundred of the Iberian Peninsula spider species are considered troglobiont or troglophile (sensu Sket 2008, Mammola et al. 2017b) and most of this knowledge comes from captures made inside caves in karstic areas (Cardoso 2012), i.e., from natural subterranean spaces in the underground that are accessible to humans. However, the MSS is an important subterranean habitat for spiders, and its study has the potential to reveal new and interesting catches, as the extensive work by the arachnologist Vlastimil Růžička in colluvial MSS (scree slopes) from the Czech Republic has shown (see Růžička 1990; Růžička and Dolanský 2016, and references therein), as well as other studies in different European regions (see, for instance, Nae 2008; Deltshev et al. 2011; Nae and Ilie 2004).

The Sierra de Guadarrama National Park, located in the Central System of the Iberian Peninsula, was recently established in order to protect the high-elevation areas and summits of the Guadarrama Mountains (BOE 2013). This mountain range has traditionally received considerable attention from scientists and naturalists, but only a few recent studies have focused on its shallow subterranean environment (see Baquero et al. 2017; Gilgado et al. 2017). The aim of the present study was to characterise, across an entire year, the diversity of spider species inhabiting the colluvial MSS present throughout the National Park and to evaluate the importance of this habitat for the conservation of rare and poorly known species.

## Materials and methods

### Study area

**Figure 1.**
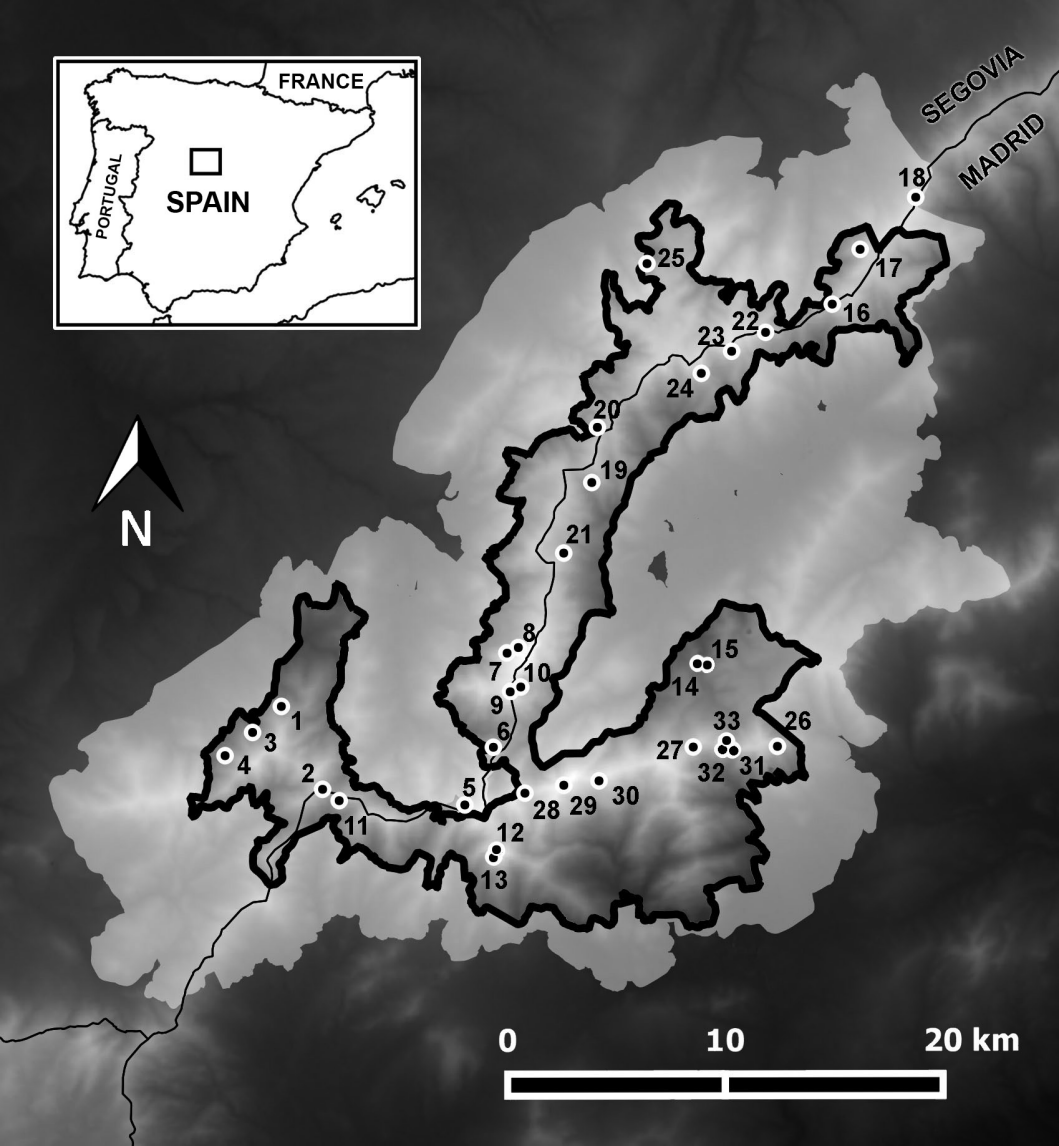
Location of the 33 scree slopes that were sampled in the Sierra de Guadarrama National Park. Each point corresponds to one subterranean sampling device (SSD). The innermost area of the map delimited by the thick black line delimits the National Park and the surrounding light gray area corresponds to the buffering zone (peripheral protection area).

Sierra de Guadarrama National Park is located in the Central System of the Iberian Peninsula, between the two provinces of Madrid and Segovia (Figure 1). It covers an expanse of 33960 hectares (BOE 2013) and is surrounded by a peripheral buffering zone of 62687 hectares (MAPAMA no year). The altitudinal gradient ranges from 1200 to 2428 m a.s.l.; consequently, the climate and vegetation show the expected variability associated with this type of gradient. Globally, the climate of Sierra de Guadarrama National Park can be categorised as cold continental Mediterranean: it has a short, dry, and chilly summer season and a long cold winter (PNSG no year). Precipitation in the highest elevations occurs mostly in the form of snow, forming a layer that lasts all winter and part of the spring season (Salazar Rincón and Vía García 2003). The orthogneiss, rocks of metamorphic quartz-feldspathic origin, are the predominant rocks of the Park (Vialette et al. 1987). The fragmentation of these rocks, mainly as a direct consequence of past (pre)glacial events, is the origin of the typical moraines and colluvial deposits (Sanz 1986), the so-called scree slopes. Baquero et al. (2017) provide more details on the different bioclimatic levels present in Guadarrama and an extensive description of the study area.

### Sampling

After evaluating the amount of effort that could be spent in the time available for field work, 33 scree slopes were selected across the Sierra de Guadarrama National Park with the intention of covering most of the geographic area of the Park, while taking into account access possibilities (Figure 1). Of these, 31 sampling localities were placed in the National Park and the other 2 were located in the peripheral cushioning area. A subterranean sampling device (SSD) was installed on each scree slope; a full description of these traps, which are designed to collect species inhabiting the MSS, can be found in Baquero et al. (2017). Briefly, each SSD is made up of a PVC cylinder one meter long and 11 cm in diameter that has numerous perforations (8 mm in diameter) from the middle to its base (50–90 cm). The cylinder is inserted vertically into an excavated hole, a pitfall baited with very smelly cheese and filled with 1,2-propanediol is placed at the bottom and the SSD is covered and made flush with the ground surface. The sampling period was from 20-V-2015 to 13-VII-2016. Sampling permits for the corresponding National Park were obtained from the following appropriate authorities: Ismael Hernández Fernández, Deputy Directorate General of Management and Planning of Protected Areas (2015) and José Lara Zabía, Head of Conservation Area of Flora and Fauna (2016) at the General Directorate of Environment of the Community of Madrid, and José Ignacio Quintanilla Rubio (2015), and Montserrat de Andrés Boal (2016), General Director of the Natural Environment by delegation of the Head of the Territorial Service of the Environment of the Junta de Castilla y León. Samples were taken to the laboratory and the spider specimens were separated from the rest of the material and stored in glass vials filled with 70% ethanol. All the samples were deposited in the collection of the University of Alcala. Adult specimens were identified to the species level using the keys and the compilation of identification work available in Nentwig et al. (2018). Species nomenclature in this study follows that of the World Spider Catalog (2018).

### Assessment of inventory completeness

The accumulation of new species as a function of sampling effort was assessed by building a sample-based species accumulation curve by randomising the order of entrance of each SSD (sample) 100 times and calculating the mean species richness for each level of sampling effort (number of SSDs; Gotelli and Colwell 2001). The following non-parametric species richness estimators were calculated: Chao1, Chao2, first-order Jackknife (Jack1), second-order Jackknife (Jack2), ICE, and ACE (Colwell and Coddington 1994) The numbers of singletons (species with one specimen), doubletons (species with two specimens), unique species (those appearing in one SSD) and duplicates (species that appear in two SSDs) were also determined. The expected number of species (Sest) for each level of sampling effort was calculated and extrapolated up to 66 samples (double the sampling effort) using the analytical approach based on the Bernoulli product moment proposed by Colwell et al. (2012). All calculations were done using EstimateS version 9.1.0 software (Colwell 2013).

### Distribution and ecotype characterisation of the species

The chorotype classification proposed by Vigna Taglianti et al. (1992) was used to classify each species following the information of the World Spider Catalog (2018) and Nentwig et al. (2018). The Iberian Spider Catalogue (Morano et al. 2014) was used as a primary consulting source, and after a thorough search of the literature up to 2018, each species was evaluated as a possible new record for either the Segovia or Madrid provinces, for the Sierra de Guadarrama National Park or for the Iberian Peninsula. Except for the most common and widespread species (see Results), the presence records in the Iberian Peninsula for each species were compiled from the literature up to 2018. All records were referred to a 10 × 10 km UTM grid; only a few records were discarded because the provided place name was insufficiently precise to be ascribed to a unique 10 × 10 km UTM cell. Distribution maps were built with QGIS version 2.16.3 software (Quantum GIS Development Team 2016).

Troglobiont or troglophile species were classified as such, following Mammola et al. (2017b). Each species was characterised as newly or previously recorded from the MSS after a literature review.

## Results

**Figure 2.**
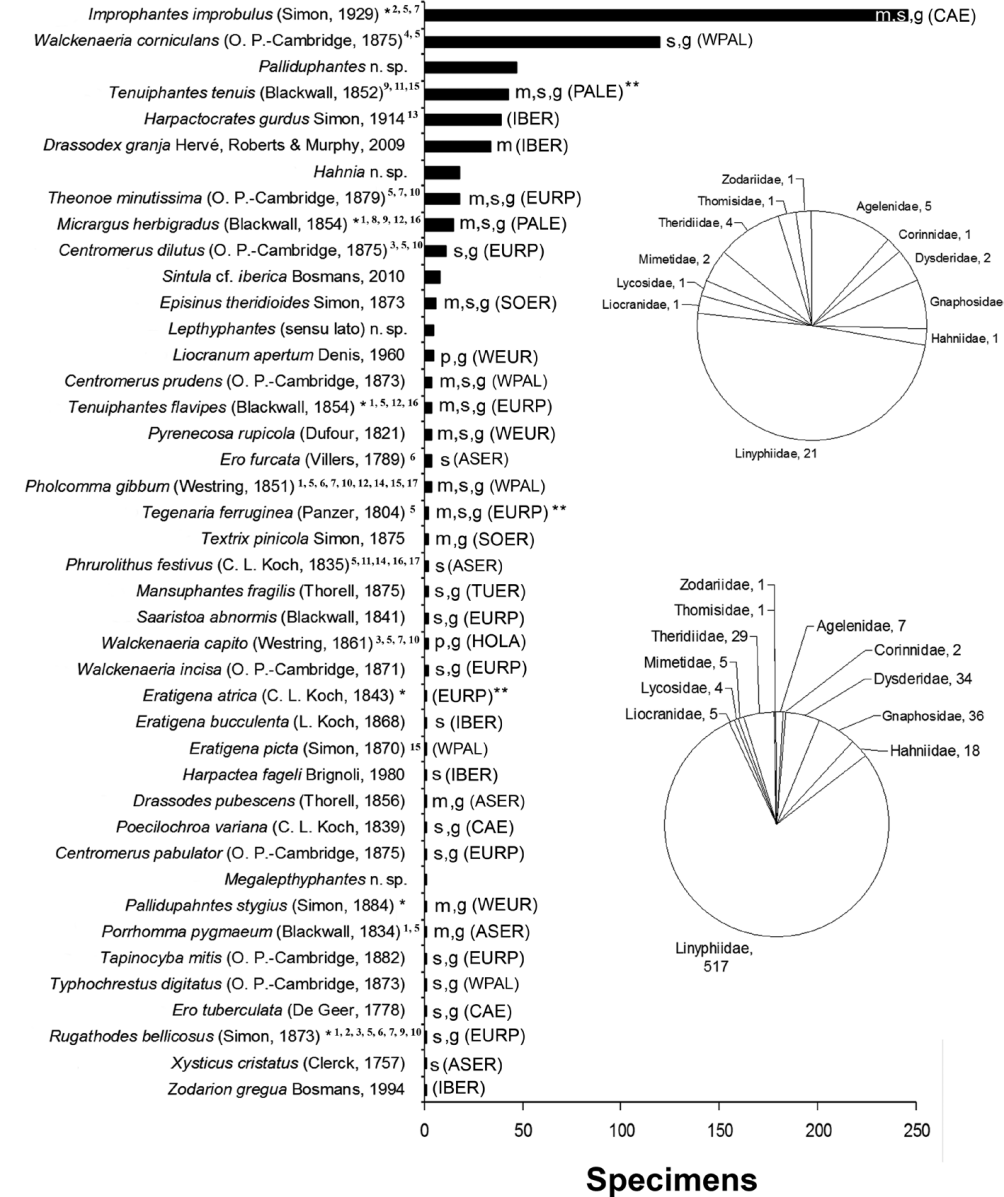
Number of specimens for each spider species. Pie charts represent the distribution of species (up) and specimens (down) into spider families in the inventory. Abbreviations: m = new species record for Madrid province; s = new species record for Segovia province; p = new species record for the Iberian Peninsula; g = new species record for the Sierra de Guadarrama National Park. Chorotypes based on the criteria proposed by Vigna Taglianti et al. (1992): (HOLA) = Holarctic; (PALE) = Palearctic; (WPAL) = West-Palearctic; (ASER) = Asiatic-European; (CAE) = Centralasiatic-European; (TUER) = Turanian-European; (EURP) = European; (SOER) = South-European; (WEUR) = West-European. The chorotype Iberian Endemism (IBER) follows the criteria of Novoa (1975) and Serrano et al. (2003). * = troglophile species (sensu Mammola et al. (2017b); ** = species introduced in other regions beyond its original range (following Nentwig et al. 2018). Records for species previously listed in the MSS are given in: ^1^Růžička (1989); ^2^Růžička (1990); ^3^Růžička and Zacharda (1994); ^4^Růžička et al. (1995); ^5^Růžička and Hajer (1996); ^6^Růžička and Thaler (2002); ^7^Růžička and Klimeš (2005); ^8^Nitzu, et al. (2006); ^9^Nitzu, et al. (2010); ^10^Růžička and Zacharda (2010); ^11^Deltshev et al. (2011); ^12^Laška et al. (2011); ^13^Barranco et al. (2013); ^14^Langourov et al. (2014); ^15^Jiménez-Valverde et al. (2015); ^16^Růžička and Dolanský (2016); ^17^Mammola et al. (2017a).

**Figure 3.**
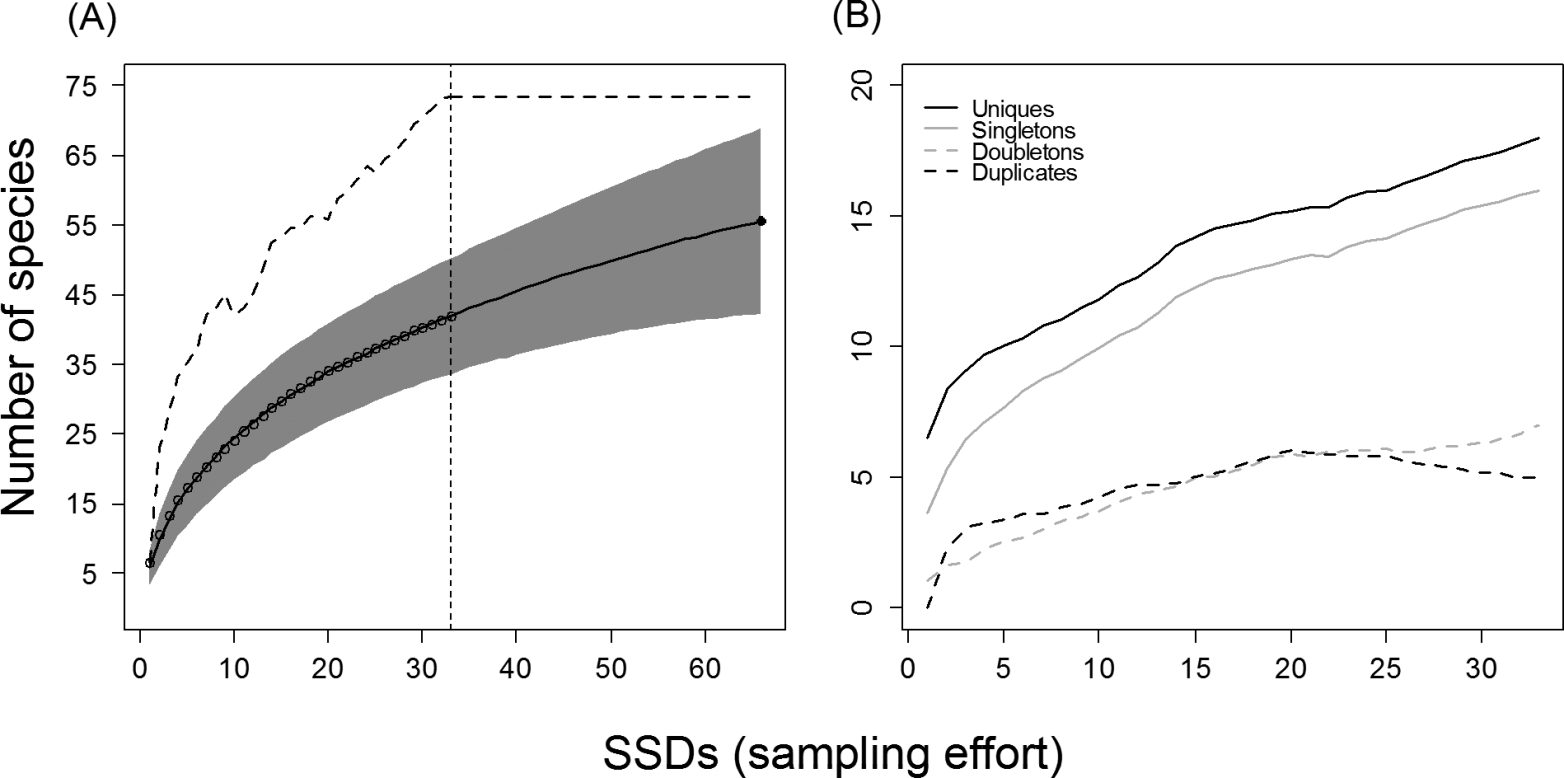
Species accumulation curves for the complete inventory of the Sierra de Guadarrama National Park. **A** Sample-based species accumulation curve using subterranean sampling devices (SSDs) as effort units (empty circles); extrapolation curve for a sampling effort of 66 SSDs (thick black line); 95% confidence interval as grey bands, and Chao2 curve (stripped line). Vertical line marks the realized sampling effort (33 SSDs) **B** Species accumulation curves of singleton, doubleton, unique and duplicate species.

In total, 1388 spiders were collected, although only 665 specimens, belonging to 42 species and 12 families, were adults (Figure 2 and Suppl. material 1: Table S1). The Linyphiidae was the most important family both in terms of species and specimens numbers. This was followed by the Theridiidae, Gnaphosidae, Dysderidae and Agelenidae (Figure 2). The accumulation curve ended while still rising (Figure 3A), as did the curves of most of the non-parametric estimators (Figure 3A and Suppl. material 2: Figure S1). The only exception was Chao1, whose curve started to stabilise at around 27 SSDs (Suppl. material 2: Figure S1). The number of singletons, doubletons, and unique species continued to increase at the end of the inventory; only the number of duplicates started to decrease (Figure 3B). Overall, 23 of 42 species (54.8%) were represented by just one or two specimens (singletons = 16, doubletons = 7) or appeared in only one or two samples (uniques = 18, duplicates = 5). The estimated degree of completeness of the inventory ranged from 57.2% to 70.6% (estimated number of species [mean ± SD]: Chao2 = 73.42 ± 21.17, Jack2 = 71.81 ± 0.00, ICE = 68.19 ± 0.03, ACE = 61.27 ± 0.00, Chao1 = 60.26 ± 12.22, Jack1 = 59.45 ± 4.42). Doubling the sampling effort (66 SSDs) predicted the addition of 14 species to the inventory (55.61 species; Figure 3A).

**Figure 4.**
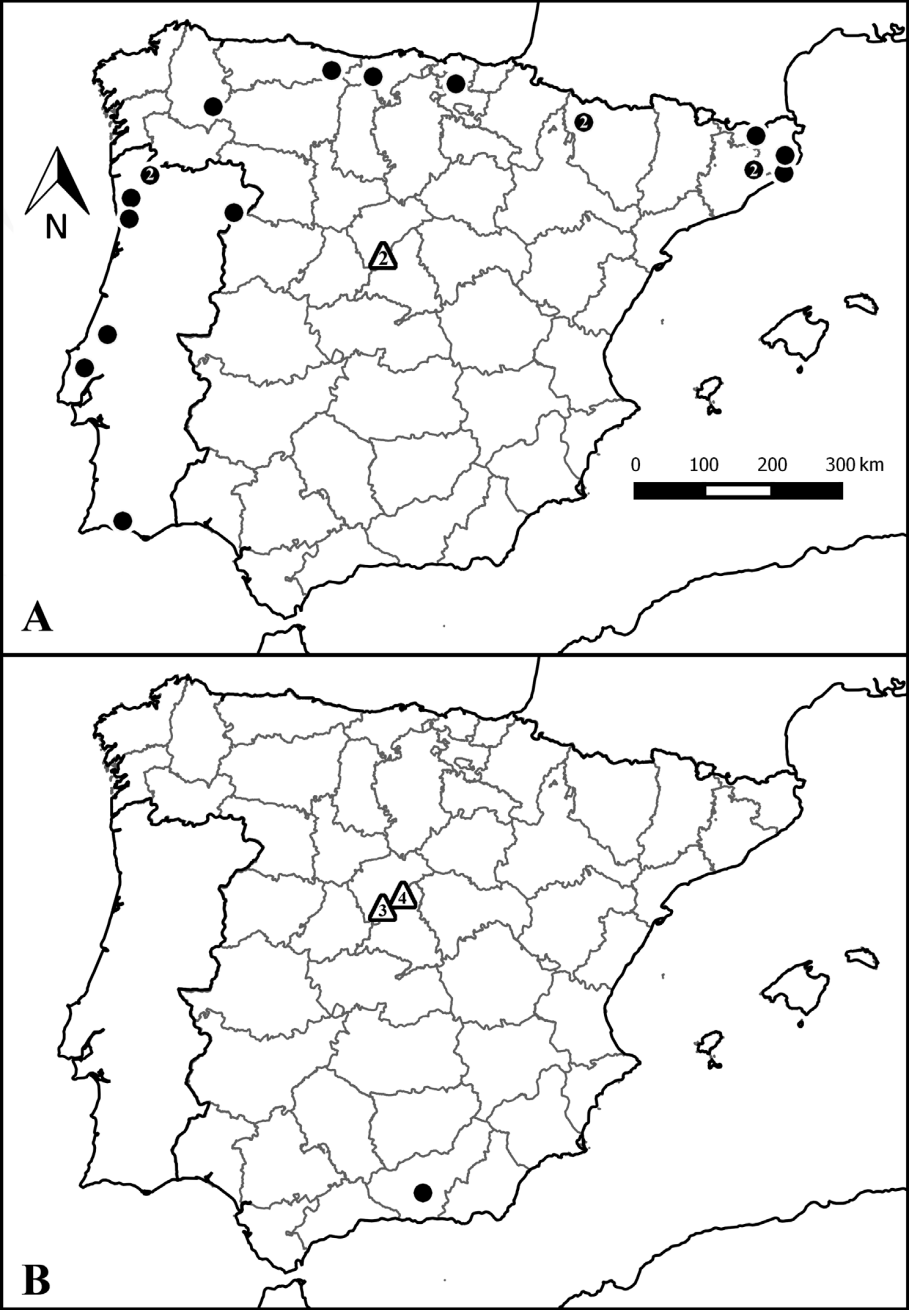
Species distribution maps. (A) *Centromerusdilutus* and (B) *Improphantesimprobulus*. Black circles represent records from the literature; triangles represent records from the present study. In cases where the symbol includes several 10 × 10 km UTM cells, and to improve legibility, their number is indicated (note that one 10 × 10 km UTM cell usually contains several sampling locations, see Suppl. material 1: Table S1).

Four species represent previously undescribed species, five species were Iberian endemics, and the remainder of the species had wider ranges of distribution (Figure 2). Almost half of the spider species captured in this study (19 out of 42) showed moderate to dramatic increases in their Iberian distribution ranges. Figure 4 illustrates two typical patterns. The first case corresponds to *Centromerusdilutus* (O. P.-Cambridge, 1875), a species of Linyphiidae that was previously only known as a restricted species in the northern and western strips of the Iberian Peninsula (Cantabrian Mountains, extending through the Pyrenees and to the west and south of Portugal; Figure 4A). The second example corresponds to *Improphantesimprobulus* (Simon, 1929), another Linyphiidae species that was only known from a locality in southern Spain (Figure 4B). The other 19 species followed similar patterns (see Suppl. material 3: Figure S2). Without considering Sintulacf.iberica and the four new species, a total of 15 species are new records for the province of Madrid, 26 species are new records for Segovia, two species are recorded for the first time in the Iberian Peninsula, and 27 species are new additions to the spider checklist of the Sierra de Guadarrama National Park (Figure 2).

One noteworthy observation is that some of the most common species in this inventory were poorly known until now. For instance, *I.improbulus* was the most numerous species (246 specimens; Figure 2) and was widely distributed across the surveyed area (24 out of 33 SSDs; Suppl. material 1: Table S1), although it was previously only known from a record published in 1957 (Janetscheck 1957) (Figure 4B). *Drassodexgranja* Hervé, Roberts & Murphy, 2009, with 34 specimens and also widely distributed across the surveyed area (15 out of 33 SSDs; Suppl. material 1: Table S1), was only known from a record dating back to 1914 (Simon 1914; Suppl. material 3: Figure S2). Two of the new species for science had 47 (*Palliduphantes* sp. nov.) and 18 (*Hahnia* sp. nov.) specimens.

Only six of 42 species were categorised as troglophile species; one of them, *I.improbulus*, was the most abundant species of the inventory (Figure 2). Four of these species plus another 12 species from the whole species list had been previously reported in other MSS studies (Figure 2).

## Discussion

The order Araneae is one of the dominant taxa in the MSS (Mammola et al. 2016), especially when the number of species is considered. On the contrary, when the number of specimens is taken into account the relevance of the taxon decreases (e.g., Jiménez-Valverde et al. 2015). This occurs because spider assemblages in the MSS tend to be very uneven and are mostly represented by only a few abundant species and a high number of extremely rare species. This pattern is confirmed in the present study, where more than half the species are represented by one or two specimens (38.1% and 16.7%, respectively). Although making comparisons is difficult due to methodological differences among studies, this number of rare species is similar to those reported in other studies across Europe; for instance, 48.6% and 11.4% in Růžička (1989), 39.1% and 13% in Růžička and Zacharda (1994), 20.8% and 14.6% in Laška et al. (1995), 46.4% and 14.3% in Růžička et al. (1995), 34.2% and 31.6% in Růžička (1996), 25.9% and 16.7% in Růžička and Hajer (1996), 52% and 12% in Růžička and Thaler (2002), 51.9% and 13.5% in Růžička and Zacharda (2010), 55% and 5% in Jiménez-Valverde et al. (2015), 42.4% and 9.1% in Růžička and Dolanský (2016) and 50% and 5.6% in Mammola et al. (2017a) (the percentage values correspond to singletons and doubletons, respectively, calculated from the data provided in the tables by the aforementioned authors).

This high component of rare species makes each sample very different from the others in terms of species composition (high percentages of uniques and duplicates; Jiménez-Valverde et al. 2015); therefore, the addition of species to the inventory is slow and constant, as the species accumulation curve of the present study shows. The species accumulation curve ends while it is still rising, as do the curves of almost all the non-parametric estimators (Fig. 3 Suppl. material 2: Figure S1), indicating that still more species are expected to be found in the colluvial MSS of the Sierra de Guadarrama National Park. If these curves are far from stabilising, then caution is mandatory when interpreting richness estimations (Melo and Froehlich 2001; Thompson et al. 2003; Thompson and Thompson 2007; Gotelli and Colwell 2001). Thus, as a conservative score, at least 18 more spider species could be expected to be found in this area. Fourteen species could be added to the inventory by doubling the number of sampled scree slopes, which would imply a significant increase in terms of monetary and work effort.

A low degree of inventory completeness is typical of hyperdiverse taxa such as spiders (Colwell and Coddington 1994; Coddington et al. 1996; Dobyns 1997; Toti et al. 2000; Sørensen et al. 2002; Scharff et al. 2003; Cardoso et al. 2008), and is even magnified in a habitat like the MSS, as the current study and other studies (e.g., Jiménez-Valverde et al. 2015) have shown. In fact, the proportion of singletons in this and other MSS studies is comparable to the percentages frequently found in spider inventories from tropical forests (see Coddington et al. 2009). As Coddington et al. (2009) suggested for tropical arthropod surveys, undersampling is probably the main cause of the high number of species represented by just one specimen in the MSS, and this is further exacerbated by the generally low densities attained in the subterranean realm (Růžička and Hajer 1996; Růžička and Klimeš 2005; Mammola et al. 2016). However, a second factor may provide an equally important explanation for the presence of low-prevalence species in the MSS. As already pointed out by Jiménez-Valverde et al. (2015), delimiting the sampling universe is extremely challenging due to the closeness to the surface and because of the ecotone role of the MSS (Moseley 2010). Thus, an unknown proportion of the rare species encountered in the present study is likely to represent simply occasional visitors, such as *Xysticuscristatus* (Clerck, 1757), *Zodariongregua* (Bosmans, 1994) or *Pyrenecosarupicola* (Dufour, 1821). These are, however, important elements (or they are at least as important as other equally rare troglophile/troglobiont species) of the interaction network in the MSS (Gers 1998; Pipan et al. 2011; Nitzu et al. 2014), and this is a good reason to consider them as part of the inventory. The high proportion of low-prevalence species in the MSS makes obtaining complete and reliable arthropod inventories a challenge, which, in turn, hampers the understanding of biodiversity and ecological patterns in the MSS (Jiménez-Valverde et al. 2015).

The presence of an important number of exogenous species in the MSS is the rule rather than the exception, and this presence will depend on the characteristics of the MSS and on the depth at which the traps are placed (Medina and Oromí 1990; Mammola et al. 2016). However, this number is difficult to estimate due to the lack of basic autecological knowledge for most of the species (the Hutchinsonian shortfall; see (Cardoso et al. 2011; Hortal et al. 2015). Following the classification proposed by Mammola et al. (2017b), which is mostly based on morphological traits, only 14.3% of the species collected in the present study can be considered troglophiles, and no troglobite species were captured. However, 28.6% of the species have been reported previously from the MSS in other studies but have not been classified as troglophiles by Mammola et al. (2017b), so their ecological preferences should probably be re-evaluated and some of them be classified as troglophiles as well. The original description of *D.granja* did not comment on any aspect of its biology and did not highlight any morphological adaptation to the subterranean environment (Simon 1914). However, the density and the extent of its occurrence in Guadarrama reported in the present study suggest a substantial affinity for the underground realm, which leads us to consider *D.granja* to be a troglophile species. The four new species also need to be classified as troglophiles. Adaptation to the subterranean environment can take many forms besides morphology (Sket 2008), and the study of the MSS will increase the knowledge about the ecological preferences of many species and will help in establishing a better ecotype classification for many of them.

Four species collected in this study were new to science. The Linnaean shortfall (i.e., the discrepancy between the number of already described species and the number of species that actually exist) manifests particularly in poorly studied habitats (Lomolino 2004; Hortal et al. 2015), as is the case for the MSS (Jiménez-Valverde et al. 2015; Mammola et al. 2016). Three of these four new species were Linyphiidae, which is the dominant spider family in subterranean habitats (Bellés 1987; Růžička 1989; Ribera and Juberthie 1994; Mammola et al. 2017a) and one of the most poorly known families (Melic et al. 2015), probably due to its small size, high diversity and difficult taxonomy.

*Liocranumapertum* Denis, 1960 and *Walckenaeriacapito* (Westring, 1861) are new species for the Iberian Peninsula. Whereas the first one has only been recorded in France, the second has a West-Palearctic distribution (World Spider Catalog 2018). More than half of the species (31 out of 42 species, 73.8%) are new records for the province of Segovia, Madrid or for both. Many of these species have wide distribution ranges (Figure 2). For instance, *I.improbulus*, with 246 specimens collected in this study, is found from Spain to China (World Spider Catalog 2018), although the single record from Spain dates back more than half a century (Janetscheck 1957). The records of most of all these species imply not only new provincial records but also significant increases in their extent of occurrence (sensu IUCN 2001) in the Iberian Peninsula. All these results evidence the scarce arachnological knowledge in the Iberian Peninsula (Melic 2001; Morano 2004; Cardoso and Morano 2010), and this significant lack of chorological information hinders not only the accurate delimitation of distributional ranges but also the accurate prediction of ranges through statistical techniques such as species distribution models (Lobo 2008). Moreover, the impossibility to precisely estimate the distribution of the species (the Wallacean shortfall; see Lomolino 2004; Hortal et al. 2015) hampers the proper application of certain criteria that are used to categorize species into different grades of vulnerability (IUCN 2001).

Usually, protected natural areas, especially those easily accessible and close to big urban areas, are highly attractive for recorders (Dennis et al. 1999; Boakes et al. 2010). Yet, the general lack of spider records for Iberian protected natural areas has been repeatedly recognized (Barriga et al. 2006; Pérez Sánchez and Méndez Iglesias 2013; Morano 2018). When it comes to National Parks, the maximum protection status, the scenario is still disheartening; despite the existence of 12 National Parks in the Iberian Peninsula, only three of them have been submitted to a relatively intense sampling effort: the Picos de Europa National Park (Méndez 1998; Pérez Sánchez and Méndez Iglesias 2013; Mardomingo Vargas et al. 2016; Jiménez Segura et al. 2017), the Cabañeros National Park (Barriga et al. 2006, 2010) and the Tablas de Daimiel National Park (Morano 2018). In the case of the Sierra de Guadarrama National Park, there is no published catalog for the area and the citations of spider species are scattered through the literature. The literature survey provided a preliminary checklist of the Park spider species and revealed that 32 (27 plus the four new species plus S.cf.iberica) out of the 42 species were new additions, which increased the list of species from 120 to 153 species. This lack of information about spiders in protected natural spaces necessarily excludes these organisms from their protection plans (Skerl and Gilliespie 1999).

## Supplementary materials

Supplementary material 1 Table S1. Araneae species captured and their distribution in the MSS of the Sierra de Guadarrama National Park Data type: species data Explanation note: UTM 1 × 1 km cells are provided and the corresponding SSD numbers (see Fig. 1) are indicated into parentheses. Note that some SSDs lay in the same UTM cell. This dataset is made available under the Open Database License (http://opendatacommons.org/licenses/odbl/1.0/). The Open Database License (ODbL) is a license agreement intended to allow users to freely share, modify, and use this Dataset while maintaining this same freedom for others, provided that the original source and author(s) are credited.Enrique Ledesma, Alberto Jiménez-Valverde, Alberto de Castro, Pablo Aguado-Aranda, Vicente M. Ortuño

Supplementary material 2 Figure S1. Non-parametric species richness estimators Data type: statistical data This dataset is made available under the Open Database License (http://opendatacommons.org/licenses/odbl/1.0/). The Open Database License (ODbL) is a license agreement intended to allow users to freely share, modify, and use this Dataset while maintaining this same freedom for others, provided that the original source and author(s) are credited.Enrique Ledesma, Alberto Jiménez-Valverde, Alberto de Castro, Pablo Aguado-Aranda, Vicente M. Ortuño

Supplementary material 3 Figure S2. Species distribution maps Data type: occurrence Explanation note: Black circles represent records form the literature; triangles represent records from the present study. In cases where the symbol includes several 10 × 10 km UTM cells, and to improve legibility, their number is indicated (note that one 10 × 10 km UTM cell usually contain several sampling locations, see Table S1). A) *Centromeruspabulator*; B) *Drassodespubescens*; C) *Episinustheridioides*; D) *Erotuberculata*; E) *Mansuphantesfragilis*; F) *Micrargusherbigradus*; G) *Pholcommagibbum*; H) *Poecilochroavariana*; I) *Pyrenecosarupicola*; J) *Rugathodesbellicosus*; K) *Saaristoaabnormis*; L) *Tapinocybamitis*; M) *Tegenariaferruginea*; N) *Tenuiphantesflavipes*; O) *Theonoeminutissima*; P) *Typhochrestusdigitatus*; Q) *Walckenaeriaincisa*. This dataset is made available under the Open Database License (http://opendatacommons.org/licenses/odbl/1.0/). The Open Database License (ODbL) is a license agreement intended to allow users to freely share, modify, and use this Dataset while maintaining this same freedom for others, provided that the original source and author(s) are credited.Enrique Ledesma, Alberto Jiménez-Valverde, Alberto de Castro, Pablo Aguado-Aranda, Vicente M. Ortuño
