# Supplementary material for: The study of hidden habitats sheds light on poorly known taxa: spiders of the Mesovoid Shallow Substratum
Source: Zookeys. 2019 Apr 23;841:39–59. doi: 10.3897/zookeys.841.33271 (PMC6495052; doi:10.3897/zookeys.841.33271)
Supplement: Supplementary material 1 [file zookeys-841-039-s001.docx]

Table S1. Araneae species captured and their distribution in the MSS of the Sierra de Guadarrama National Park. UTM 1x1 km cells are provided and the corresponding SSD numbers (see Fig. 1) are indicated into parentheses. Note that some SSDs lay in the same UTM cell.

| **Family** | **Species** | **UTM 1 × 1 km** |
| --- | --- | --- |
| **Agelenidae** | *Eratigena atrica* | 30TVL2127 (21) |
|  | *Eratigena bucculenta* | 30TVL0518 (4) |
|  | *Eratigena picta* | 30TVL2440 (25) |
|  | *Tegenaria ferruginea* | 30TVL1615 (5); 30TVL2818 (33) |
|  | *Textrix pinicola* | 30TVL2116 (29); 30TVL2230 (19) |
| **Corinnidae** | *Phrurolithus festivus* | 30TVL2127 (21); 30TVL2440 (25) |
| **Dysderidae** | *Harpactea fageli* | 30TVL1016 (11) |
|  | *Harpactocrates gurdus* | 30TVL0518 (4); 30TVL0619 (3); 30TVL1016 (2,11); 30TVL1713 (13); 30TVL1822 (7); 30TVL1916 (28); 30TVL1923 (8); 30TVL2230 (19); 30TVL2233 (20); 30TVL2440 (25); 30TVL2818 (33); 30TVL2836 (23); 30TVL3018 (26); 30TVL3338 (16); 30TVL3743 (18) |
| **Gnaphosidae** | *Drassodes pubescens* | 30TVL1713 (13) |
|  | *Drassodex granja* | 30TVL1016 (2); 30TVL1615 (5); 30TVL1813 (12); 30TVL2116 (29); 30TVL2127 (21); 30TVL2217 (30); 30TVL2718 (27); 30TVL2818 (5,6); 30TVL2230 (19); 30TVL2233 (20); 30TVL3018 (26); 30TVL3037 (22); 30TVL3338 (16); 30TVL3743 (18) |
|  | *Poecilochroa variana* | 30TVL0619 (3) |
| **Hahniidae** | *Hahnia* n. sp. | 30TVL0518 (4); 30TVL0619 (3); 30TVL1615 (5); 30TVL1718 (6); 30TVL2217 (30); 30TVL2440 (25); 30TVL2718 (27); 30TVL2818 (31,33); 30TVL3018 (26) |
| **Linyphiidae** | *Centromerus dilutus* | 30TVL0518 (4); 30TVL0619 (3); 30TVL0820 (1); 30TVL1016 (1); 30TVL1923 (8); 30TVL3338 (16) |
|  | *Centromerus pabulator* | 30TVL2440 (25) |
|  | *Centromerus prudens* | 30TVL2127 (21); 30TVL2722 (4); 30TVL2818 (6); 30TVL3441 (17) |
|  | *Improphantes improbulus* | 30TVL0518 (4); 30TVL0619 (3); 30TVL1016 (11); 30TVL1615 (5); 30TVL1713 (13); 30TVL1813 (12); 30TVL1821 (9); 30TVL1822 (7); 30TVL1916 (28); 30TVL1923 (8); 30TVL2116 (29); 30TVL2217 (30); 30TVL2230 (19); 30TVL2233 (20); 30TVL2718 (27); 30TVL2722 (14,15); 30TVL2818 (31,33);  30TVL2836 (23); 30TVL3037 (22); 30TVL3338 (16); 30TVL3441 (17); 30TVL3743 (18) |
|  | *Lepthyphantes* (sensu lato) n. sp. | 30TVL0820 (1); 30TVL1016(2); 30TVL2440 (25) |
|  | *Mansuphantes fragilis* | 30TVL1718 (6) |
|  | *Megalepthiphantes* n. sp. | 30TVL0619 (3) |
|  | *Micrargus herbigradus* | 30TVL0518 (4); 30TVL0820 (1); 30TVL1016(2); 30TVL2217 (30); 30TVL3441 (17); 30TVL3743 (18) |
|  | *Palliduphantes stygius* | 30TVL2836 (23) |
|  | *Palliduphantes* n. sp. | 30TVL0518 (4); 30TVL0619 (3); 30TVL1016(2,11); 30TVL2440 (25); 30TVL2836 (23); 30TVL3018 (26); 30TVL3037 (22); 30TVL3338 (16) |
|  | *Porrhomma pygmaeum* | 30TVL2836 (23) |
|  | *Saaristoa abnormis* | 30TVL3743 (18) |
|  | *Sintula* cf. *iberica* | 30TVL0619 (3); 30TVL0820 (1); 30TVL1718 (6) |
|  | *Tapinocyba mitis* | 30TVL1718 (6) |
|  | *Tenuiphantes flavipes* | 30TVL0518 (4); 30TVL1813 (12); 30TVL1821 (9); 30TVL2440 (25) |
|  | *Tenuiphantes tenuis* | 30TVL0518 (4); 30TVL0619 (3); 30TVL1016 (2); 30TVL1615 (5); 30TVL1713 (13); 30TVL1813 (12); 30TVL1822 (7); 30TVL1916 (28); 30TVL1923 (8); 30TVL2116 (29); 30TVL2217 (30); 30TVL2233 (20); 30TVL2440 (25); 30TVL2722 (15); 30TVL2818 (31,33); 30TVL3037 (22); 30TVL3743 (18) |
|  | *Typhochrestus digitatus* | 30TVL3338 (16) |
|  | *Walckenaeria capito* | 30TVL3441 (17); 30TVL3743 (18) |
|  | *Walckenaeria corniculans* | 30TVL0518 (4); 30TVL0619 (3); 30TVL0820 (1); 30TVL1713 (13); 30TVL1718 (6); 30TVL1813 (12); 30TVL1821 (9); 30TVL1822 (7); 30TVL1916 (28); 30TVL1923 (8); 30TVL2116 (29); 30TVL2230 (19); 30TVL2718 (27); 30TVL2818 (31,33); 30TVL2836 (23); 30TVL3018 (26); 30TVL3037 (22); 30TVL3441 (17); 30TVL3743 (18) |
|  | *Walckenaeria incisa* | 30TVL1016 (2); 30TVL1718 (6) |
| **Liocranidae** | *Liocranum apertum* | 30TVL0518 (4); 30TVL1822 (7); 30TVL2440 (25) |
| **Lycosidae** | *Pyrenecosa rupicola* | 30TVL0518 (4); 30TVL2217 (30); 30TVL2230 (19); 30TVL3018 (26) |
| **Mimetidae** | *Ero furcata* | 30TVL0820 (1); 30TVL1615 (5); 30TVL3441 (17); 30TVL3743 (18) |
|  | *Ero tuberculata* | 30TVL0518 (4) |
| **Theridiidae** | *Episinus theridioides* | 30TVL1615 (5); 30TVL1813 (12); 30TVL1921 (10); 30TVL2818 (31) |
|  | *Pholcomma gibbum* | 30TVL1016 (11); 30TVL1822 (7); 30TVL2440 (25) |
|  | *Rugathodes bellicosus* | 30TVL2233 (20) |
|  | *Theonoe minutissima* | 30TVL0518 (4); 30TVL0619 (3); 30TVL0820 (1); 30TVL1016 (1,2); 30TVL1615 (5); 30TVL2230 (19); 30TVL2440 (25); 30TVL2722 (15); 30TVL2818 (31); 30TVL3018 (26); 30TVL3338 (16) |
| **Thomisidae** | *Xysticus cristatus* | 30TVL3441 (17) |
| **Zodariidae** | *Zodarion gregua* | 30TVL2127 (21) |
