# Supplementary figures and images for: The study of hidden habitats sheds light on poorly known taxa: spiders of the Mesovoid Shallow Substratum
Source: Zookeys. 2019 Apr 23;841:39–59. doi: 10.3897/zookeys.841.33271 (PMC6495052; doi:10.3897/zookeys.841.33271)

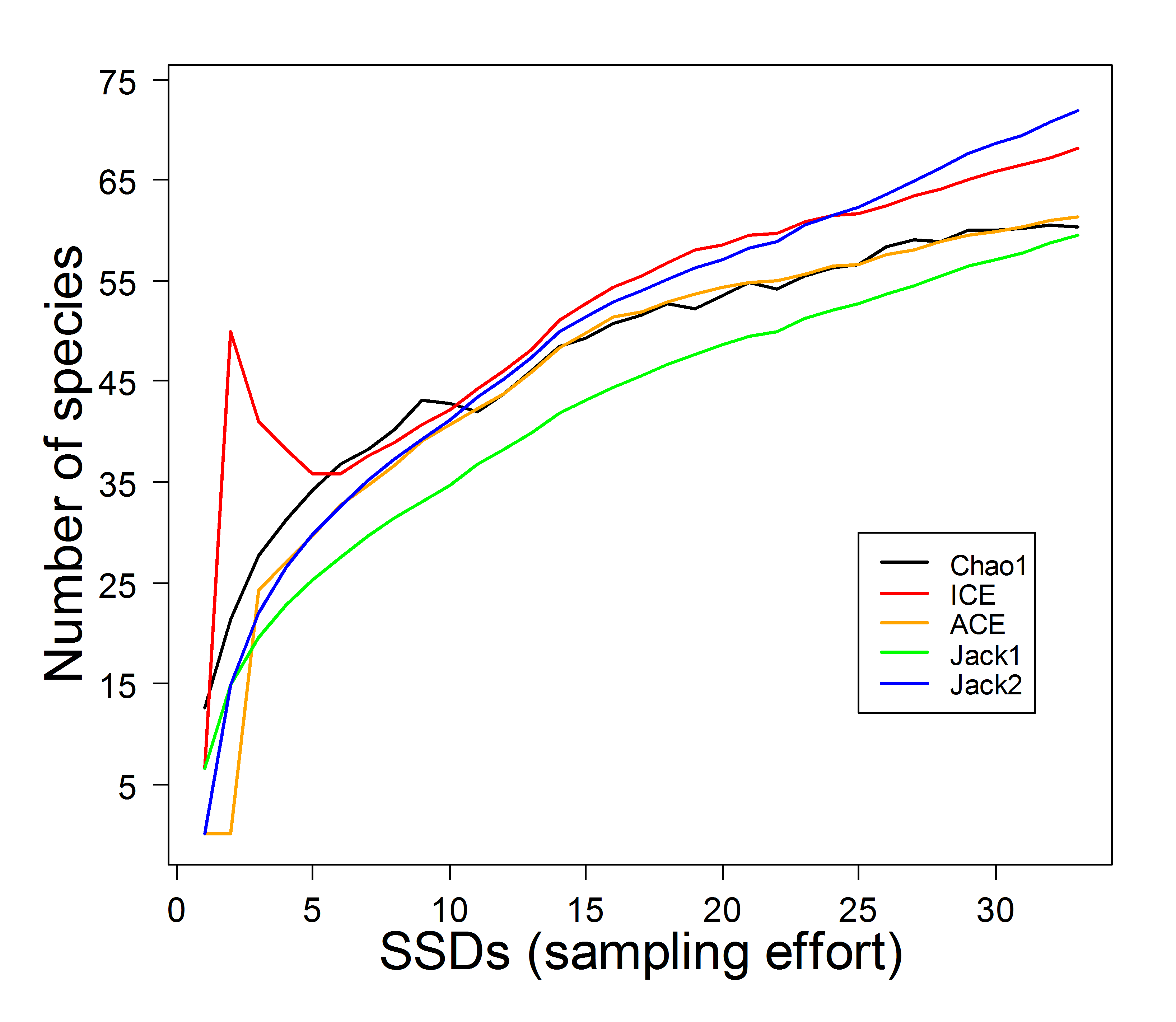

Supplement: Supplementary material 2 [file zookeys-841-039-s002.tif]
